# Using a competitive counterflow assay to identify novel cationic substrates of OATP1B1 and OATP1B3

**DOI:** 10.3389/fphys.2022.969363

**Published:** 2022-09-08

**Authors:** Regina D. Schnegelberger, Brianna Steiert, Philip J. Sandoval, Bruno Hagenbuch

**Affiliations:** Department of Pharmacology, Toxicology and Therapeutics, The University of Kansas Medical Center, Kansas City, KS, United States

**Keywords:** organic anion, organic cation, CCF, transport, liver

## Abstract

OATP1B1 and OATP1B3 are two drug transporters that mediate the uptake of multiple endo- and xenobiotics, including many drugs, into human hepatocytes. Numerous inhibitors have been identified, and for some of them, it is not clear whether they are also substrates. Historically radiolabeled substrates or LC-MS/MS methods were needed to test for transported substrates, both of which can be limiting in time and money. However, the competitive counterflow (CCF) assay originally described for OCT2 and, more recently, for OCT1, OATP2B1, and OATP1A2 does not require radiolabeled substrates or LC-MS/MS methods and, as a result, is a more cost-effective approach to identifying substrates of multidrug transporters. We used a CCF assay based on the stimulated efflux of the common model substrate estradiol-17β-glucuronide (E17βG) and tested 30 compounds for OATP1B1- and OATP1B3-mediated transport. Chinese Hamster Ovary (CHO) cells stably expressing OATP1B1 or OATP1B3 were preloaded with 10 nM [^3^H]-estradiol-17β-glucuronide. After the addition of known substrates like unlabeled estradiol-17β-glucuronide, estrone-3-sulfate, bromosulfophthalein, protoporphyrin X, rifampicin, and taurocholate to the outside of the preloaded CHO cells, we observed efflux of [^3^H]-estradiol-17β-glucuronide due to exchange with the added compounds. Of the tested 30 compounds, some organic cation transporter substrates like diphenhydramine, metformin, and salbutamol did not induce [^3^H]-estradiol-17β-glucuronide efflux, indicating that the two OATPs do not transport them. However, 22 (for OATP1B1) and 16 (for OATP1B3) of the tested compounds resulted in [^3^H]-estradiol-17β-glucuronide efflux, suggesting that they are OATP substrates. Among these compounds, we further tested clarithromycin, indomethacin, reserpine, and verapamil and confirmed that they are substrates of the two OATPs. These results demonstrate that the substrate spectrum of the well-characterized organic anion transporting polypeptides includes several organic cations. Furthermore, as for other drug uptake transporters, the CCF assay is an easy-to-use screening tool to identify novel OATP substrates.

## Introduction

The organic anion transporting polypeptides OATP1B1 and OATP1B3 are multispecific transporters expressed selectively in human hepatocytes. They are important drug uptake systems and transport a wide variety of structurally diverse compounds (Hagenbuch and Stieger, 2013). Frequently the commercially available [^3^H]-estradiol-17β-glucuronide is used as a model substrate to characterize the transport of both OATPs. Numerous chemicals have been reported to interact with the two liver-specific OATP1B1 and OATP1B3 (Karlgren et al., 2012; De Bruyn et al., 2013). However, for many of these inhibitors, it is not clear whether they are also substrates. Researchers have used radiolabeled substrates or mass spectrometry to determine whether chemical compounds are substrates of OATP1B1 and OATP1B3. However, these assays are expensive and time-consuming because not all inhibitors are available in a radiolabeled form. Additionally, these assays cannot easily be performed in a medium-to high-throughput format.

In 2013, Harper and Wright (Harper and Wright, 2013) described the competitive counterflow (CCF) assay as an alternative to radiolabeled or LC/MS approaches for determining if an inhibitor is also a substrate of drug transporters. In this assay, cells that express the transporter of interest are incubated with a low concentration of a readily available radiolabeled substrate until the uptake of this substrate reaches a steady state where its influx into the cell is equivalent to its efflux. Once this constant state is reached, the cells are washed and incubated with the same substrate concentration (control) or the same substrate concentration in addition to a test compound. After a specific time, radioactivity remaining within the cells is determined. If the test compound is not transported, then the radioactivity within the cells will not change. Alternatively, if the test compound is a substrate, its uptake will drive the efflux of the radioactive substrate reducing the radioactivity in the cells. The advantage of this assay is that multiple compounds can be tested with a single readily available radioactive substrate without the need for expensive custom radiolabeled compound synthesis. Using this assay, several novel substrates for the human organic cation transporter 1 (OCT1) (Boxberger et al., 2018), the human OATP2B1 (Schafer et al., 2018), and the human OATP1A2 (Schafer et al., 2022) were identified. In the present study, we used the CCF assay for OATP1B1 and OATP1B3 using [^3^H]-estradiol-17β-glucuronide as the probe substrate and tested 27 OATP inhibitors and 3 negative controls to identify novel substrates for both OATPs.

## Materials and methods

### Materials

Radiolabeled [^3^H]-estradiol-17β-glucuronide (41.4 Ci/mmol) and [^3^H]-verapamil (83.3 Ci/mmol) were purchased from PerkinElmer (Boston, MA). [^3^H]-clarithromycin (12.2 Ci/mmol) and [^3^H]-reserpine (10 Ci/mmol) were obtained from Moravek Biochemicals (Brea, CA). [^3^H]-indomethacin (5 Ci/mmol) was purchased from American Radiolabeled Chemicals (St. Louis, MO). Unlabeled compounds were purchased from Sigma-Aldrich (St. Louis, MO); unlabeled compound 6 (3,7-(dihydroxy)-2-(3,4-dihydroxy phenyl)-5-propyloxy-4-oxo-4H-chromene) was described before (Zhang et al., 2013). All other chemicals and reagents were of analytical grade and were readily available from commercial sources.

### Cell culture and uptake experiments

Chinese Hamster Ovary (CHO) cells stably expressing OATP1B1*1b or OATP1B3 haplotype 1 were cultured as described before (Gui et al., 2008). Uptake of radiolabeled substrates was performed 24 h after induction of the cells with sodium butyrate with correction for protein concentration (determined with leftover lysates not used for scintillation counting for the time dependent uptake shown in Figure 3) as previously described on 24-well plates (Gui et al., 2008).

### Competitive counterflow assay

For the competitive counterflow assay, OATP1B1- and OATP1B3-expressing cells were preloaded on 96-well plates at room temperature for 2 h with 100 μl of 10 nM [^3^H]-estradiol-17β-glucuronide in uptake buffer (116.4 mM NaCl, 5.3 mM KCl, 1 mM NaH_2_PO_4_, 0.8 mM MgSO_4_, 5.5 mM D-glucose and 20 mM HEPES, pH adjusted to 7.4 with Tris). (Gui et al., 2008). Then efflux was initiated by the addition of 1 µl of a stock solution of 100 μM, 1 mM, or 10 mM test compound dissolved in DMSO, or DMSO for the control condition (final DMSO concentration was 1%) using a Biomek FX robot (Beckman Coulter Inc., Brea, CA). After 30 min, efflux was stopped by washing the wells three times with 100 µl of room temperature uptake solution with a Wellwash™ Versa microplate washer (Thermo Fisher Scientific Inc., Waltham, MA); cells were solubilized with 150 μl of Triton X-100. One hundred microliters of this solution were transferred to 96-well Flexible PET Microplates (PerkinElmer). After adding 200 μl of Optiphase HiSafe, samples were counted using a MicroBeta microplate scintillation counter (PerkinElmer).

### Calculations and statistics

All calculations and statistical analyses were performed using Prism 9 (GraphPad Software Inc., San Diego, CA). Significance was determined using one-way ANOVA followed by Dunnett’s multiple comparisons. Results were considered significantly different at *p* < 0.05.

## Results

### Establishing the conditions for the competitive counterflow assay using OATP1B1- and OATP1B3-expressing CHO cells

**FIGURE 1:**
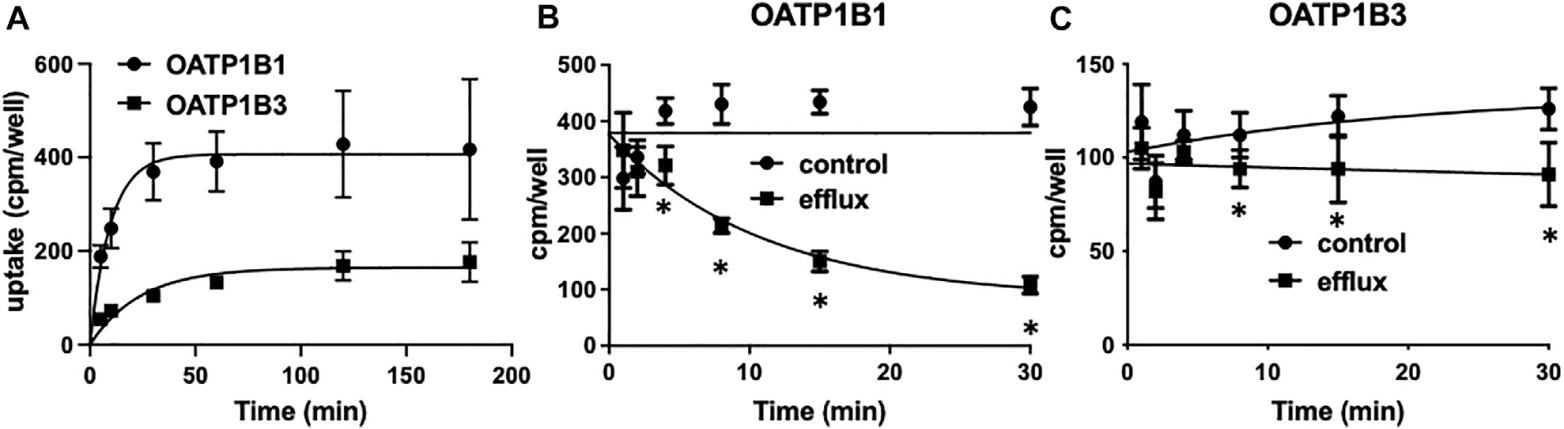
OATP1B1- and OATP1B3-mediated time-dependent uptake **(A)** and efflux **(B,C)** of estradiol-17β-glucuronide using stable CHO cell lines. **(A)** Uptake of 10 nM [^3^H]-estradiol-17β-glucuronide into CHO cells stably expressing OATP1B1 (circles) or OATP1B3 (squares) was determined at room temperature over 180 min on 96-well plates. Values are means ± SD of three independent experiments. **(B,C)** After preloading the cells for 2 h with 10 nM [^3^H]- estradiol-17β-glucuronide efflux was initiated by the addition of 100 µM unlabeled estradiol-17β-glucuronide. Values are means ± SD of two independent experiments combined with 7-8 technical replicates. *p < 0.05 as compared to the control.

To perform the competitive counterflow assay, steady-state conditions for the respective transporter must first be established. Therefore, we measured the uptake of 10 nM [^3^H]-estradiol-17β-glucuronide into CHO cells stably expressing OATP1B1 or OATP1B3 on 96-well plates at room temperature over 3 h. As shown in Figure 1A, uptake of estradiol-17β-glucuronide was slower for OATP1B3 than for OATP1B1, and steady-state was about 2.5 fold lower. Both cell lines reached steady-state at 2 h. Next, we determined time-dependent efflux from the preloaded CHO cells. OATP1B1- and OATP1B3-expressing CHO cells were loaded with 10 nM [^3^H]-estradiol-17β-glucuronide for 2 h, and then efflux was initiated by the addition of 100 μM estradiol-17β-glucuronide (efflux) or an equal volume of water (control). Figures 1B,C show that significant efflux was observed at 4 min for OATP1B1 and 8 min for OATP1B3, but the largest signals were seen at 30 min. Therefore, we determined efflux after a 30-min incubation in the presence of the potential substrate for the following experiments.

### Validating the competitive counterflow assay with known OATP substrates

**FIGURE 2:**
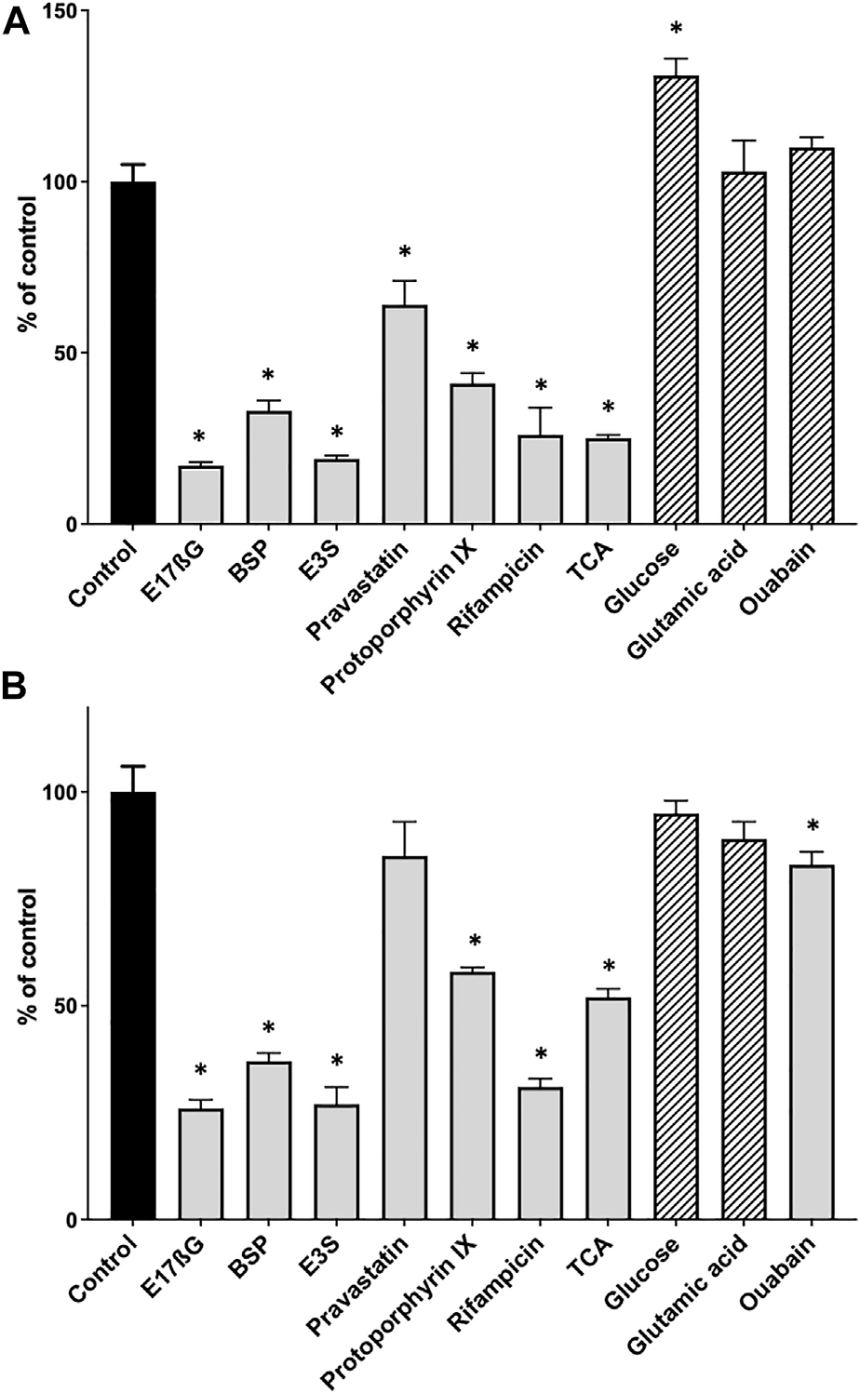
Positive and negative control substrates for the competitive counterflow assay in OATP1B1- and OATP1B3-expressing CHO cells. After cells expressing OATP1B1 **(A)** or OATP1B3 **(B)** were preloaded with 10 nM [^3^H]-estradiol-17β-glucuronide for 2 h at room temperature, concentrated solutions of known OATP substrates (positive controls, grey bars) and compounds that should not interact with OATPs (negative controls, hatched bars) were added to yield final concentrations of 100 µM. After a 30-min incubation at room temperature, cell-associated radioactivity was measured and is presented as % of control (which did not get any compound treatment). Values are mean ± SD of quadruplicate determinations in a single experiment. *p < 0.05 as compared to the control.

After establishing the basic conditions, we tested the assay using several known OATP substrates and three compounds that should not result in [^3^H]-estradiol-17β-glucuronide efflux. Figure 2A shows that the known OATP1B1 substrates estradiol-17β-glucuronide (E17βG), bromosulfophthalein (BSP), estrone-3-sulfate (E3S), pravastatin, protoporphyrin IX, rifampicin and taurocholate, all initiated the efflux of [^3^H]-estradiol-17β-glucuronide. The negative controls, glucose, glutamic acid, and ouabain (for OATP1B1), did not result in the efflux of [^3^H]-estradiol-17β-glucuronide. Glucose even resulted in a slight but significant increase of [^3^H]-estradiol-17β-glucuronide. For OATP1B3, the results were similar (Figure 2B) except for pravastatin, which did not result in a significant efflux of [^3^H]-estradiol-17β-glucuronide, and ouabain which resulted in a small but significant efflux of [^3^H]-estradiol-17β-glucuronide.

### Application of the competitive counterflow assay to identify novel OATP substrates

To identify OATP1B1 and OATP1B3 substrates, we used the competitive counterflow assay and tested 30 compounds. The original paper suggested that the compounds to be tested should be applied at ten times the IC_50_ concentration (Harper and Wright, 2013). However, given that many of our compounds have limited solubility at ten times the IC_50_ concentration, we decided to test them at three different concentrations, including 1, 10, and 100 μM. As a positive control, estradiol-17β-glucuronide was included and showed a concentration-dependent efflux (Tables 1, 2). We identified compounds that did not result in any [^3^H]-estradiol-17β-glucuronide efflux, compounds that only resulted in [^3^H]-estradiol-17β-glucuronide efflux at the highest concentration of 100 μM, compounds that showed an excellent concentration-dependent efflux, and a few chemicals that resulted in stimulation of [^3^H]-estradiol-17β-glucuronide uptake, rather than efflux (Tables 1 and 2). The “neutral” compounds for OATP1B1 were the organic cation transporter substrates diphenhydramine, metformin, and salbutamol (Table 1). The mean of these neutral compounds was used as the control value to perform statistics for the efflux in Table 1. We considered 22 compounds as OATP1B1 substrates because they resulted in a concentration-dependent efflux of [^3^H]-estradiol-17β-glucuronide from OATP1B1-expressing cells (Table 1). They include the known OATP substrates cyclosporine and resveratrol, and 20 compounds that have not been reported as OATP1B1 substrates to the best of our knowledge. We saw the most efflux with *β*-estradiol 3-sulfate, cyclosporine, bromocriptine, salinomycin, clotrimazole, cilnidipine, compound 6 (Zhang et al., 2013), and ketoconazole. There were essentially no differences in efflux between the 100 μM and the 10 μM concentration. The next group of compounds elicited different levels of efflux for the three concentrations used. This group includes progesterone, indomethacin, reserpine, DIDS, clobetasol, clarithromycin, verapamil, and gemfibrozil (Table 1). The following four compounds only resulted in apparent efflux at the highest concentration of 100 μM: pararosaniline, berberine, resveratrol, and naringin. Diosmethin and oleanolic acid resulted in significant efflux at the 1 μM concentration. However, they did not reach total efflux at the 10 μM and 100 μM concentrations, potentially due to their very low solubility in aqueous solutions. The last five compounds produced efflux with an unexpected pattern. Gramicidin, an ionophore, resulted in the same efflux at all three concentrations but did not reach the maximal efflux seen with, e.g., *β*-estradiol 3-sulfate. Gliquidone and ursolic acid resulted in less efflux at 100 μM than at 10 μM or at 1 μM, and nifedipine resulted in some efflux. Cimetidine only showed efflux at the 100 μM concentration but resulted in a stimulation of uptake at 10 μM and had no effect at 1 μM.

**TABLE 1:** Concentration-dependent efflux of [^3^H]-estradiol-17β-glucuronide from OATP1B1-expressing CHO cells, stimulated by common OATP inhibitors.

Compound	100 μM	10 μM	1 μM
*Estradiol-17β-glucuronide*	**20.3 ± 3.4**	**29.7 ± 3.7**	**63.2 ± 7.5**
Diphenhydramine	87.6 ± 10.4	96 ± 4.9	95.8 ±7.7
Metformin	93 ± 8.7	110.5 ± 7.3	111.5 ± 4.1
Salbutamol	98.2 ± 12.5	95.7 ± 12.1	94.8 ± 10.6
*β-Estradiol 3-sulfate*	**11 ± 1.0**	**10.6 ± 1.3**	**29.6 ± 11.8**
*Cyclosporine*	**15.8 ± 3.0**	**13.5 ± 2.7**	**60.9 ± 37.1**
*Bromocriptine*	**19.8 ± 0.6**	**14.6 ± 1.4**	**49.8 ± 5.5**
*Salinomycin*	**24.9 ± 1.4**	**19.9 ± 1.6**	**61.7 ± 4.9**
*Clotrimazole*	**30.5 ± 2.9**	**25 ± 2.3**	89.1 ± 34.3
*Cilnidipine*	**17.3 ± 2.1**	**25.2 ± 3.1**	81.5 ± 6.7
*Compound 6*	**26.9 ± 3.9**	**26.9 ± 5.6**	74.3 ± 26.3
*Ketoconazole*	**25.1 ± 3.5**	**28.1 ± 9**	94.9 ± 13.7
*Progesterone*	**16.9 ± 4.9**	**32.5 ± 4.9**	97.2 ± 4.3
*Indomethacin*	**25.7 ± 4.1**	**41.7 ± 7**	84.6 ± 18
*Reserpine*	**33.4 ± 3.9**	**54 ± 8**	96.3 ± 7.6
*DIDS*	**30.4 ± 8.1**	**57.2 ± 8.1**	96.3 ± 14.6
*Clobetasol*	**21.8 ± 4.3**	**63.9 ± 8.6**	110 ± 12.4
*Clarithromycin*	**20.9 ± 1.8**	**65.5 ± 6.6**	101.5 ± 7.8
*Verapamil*	**20 ± 2.3**	**68.8 ± 8.9**	100.4 ± 4.6
*Gemfibrozil*	**19 ± 3**	**69.7 ± 10.7**	99.4 ± 11.6
*Pararosaniline*	**36.8 ± 4.3**	81.8 ± 12.2	92.2 ± 10
*Berberine*	**41.4 ± 5.1**	88.4 ± 10.6	97.9 ± 12.7
*Resveratrol*	**18.3 ± 4.5**	**61.2 ± 11.3**	78.1 ± 17.3
*Naringin*	**30.9 ± 7.4**	**66.9 ± 10.6**	77.5 ± 9.3
Diosmetin*	**26.9 ± 4.8**	**33.4 ± 9.6**	**60.1 ± 11.2**
Oleanolic Acid*	**37.2 ± 3.8**	**35.8 ± 5.2**	**52.4 ± 14.8**
Gramicidin	**39.3 ± 7.4**	**29.4 ± 6.6**	**32.5 ± 5.6**
Gliquidone	**78.9 ± 7.4**	**36.8 ± 1.9**	**44.7 ± 4.6**
Ursolic acid	**68.3 ± 12.3**	**50.7 ± 7**	**63.2 ± 9.2**
Nifedipine	**67.2 ± 2.3**	**76 ± 5.9**	87.8 ± 11.9
Cimetidine	**34.3 ± 7.3**	**130.9 ± 27.3**	107.7 ± 12.7

OATP1B1-expressing CHO cells were preloaded for 2 h at room temperature with 10 nM [^3^H]-estradiol-17β-glucuronide. The potential OATP-substrates were added to yield final concentrations of 100, 10, or 1 µM and radioactivity remaining in the cells was measured after a 30-min incubation. Values are presented as % of control and represent the mean ± SD, of three independent experiments, each with quadruplicate determinations. Values in bold indicate significant differences to the control at *p* < 0.05. Compounds in italics are substrates; * compounds that are considered to be substrates.

**TABLE 2:** Concentration-dependent efflux of [^3^H]-estradiol-17β-glucuronide from OATP1B3-expressing CHO cells, stimulated by common OATP inhibitors.

Compound	100 μM	10 μM	1 μM
*Estradiol-17β-glucuronide*	**34.7 ± 11.0**	**62.8 ± 8.9**	**81.1 ± 10.4**
Diphenhydramine	84.7 ± 24.6	89.9 ± 15.2	90.2 ±17.1
Metformin	92.2 ± 5.1	100.1 ± 5.1	104.5 ± 6.1
Salbutamol	97.9 ± 20.6	92.6 ± 14.9	90.5 ± 12.0
Berberine	88.6 ± 2.2	101.4 ± 0.6	94.8 ± 8.3
*Cyclosporine*	**34.2 ± 4.7**	**35.0 ± 9.1**	**58.0 ± 22.9**
*Salinomycin*	**41.5 ± 2.4**	**41.0 ± 0.6**	**62.4 ± 5.4**
*Bromocriptine*	**41.8 ± 3.4**	**46.9 ± 6.0**	91.7 ± 4.6
*DIDS*	**44.5 ± 15.1**	**47.8 ± 8.6**	97.7 ± 16.5
*Ketoconazole*	**46.1 ± 12.3**	56.9 ± 2.2	84.7 ± 1.3
*Clobetasol*	**30.2 ± 1.5**	**44.9 ± 2.9**	92.3 ± 5.8
*β-Estradiol 3-sulfate*	**36.0 ± 2.5**	71.3 ± 5.0	96.7 ± 10.0
*Progesterone*	**32.5 ± 4.3**	76.6 ± 7.2	105.6 ± 14.5
*Indomethacin*	**45.9 ± 12.4**	79.3 ± 3.6	101.6 ± 7.7
*Cilnidipine*	**42.2 ± 3.4**	81.3 ± 0.5	94.1 ± 5.8
*Naringin*	**50.4 ± 12.3**	81.8 ± 16.2	87.2 ± 20.0
*Verapamil*	**47.6 ± 8.8**	87.7 ± 7.3	93.6 ± 10.3
*Resveratrol*	**47.4 ± 17.5**	120.3 ± 13.8	86.2 ± 12.6
Reserpine*	54.6 ± 6.3	89.2 ± 2.9	97.5 ± 4.2
Clarithromycin*	54.4 ± 4.6	98.5 ± 5.9	96.4 ± 8.2
*Cimetidine*	**49.2 ± 17.4**	105.6 ± 13.7	98.4 ± 12.7
Gemfibrozil*	57.0 ± 3.0	123.8 ± 4.0	102.9 ± 5.4
Gramicidin	59.0 ± 3.7	59.1 ± 2.5	71.5 ± 4.8
Oleanolic Acid	57.4 ± 10.4	58.9 ± 18.4	68.3 ± 12.0
Nifedipine	77.6 ± 8.0	**175.9 ± 12.7**	114.4 ± 11.9
Ursolic acid	**135.7 ± 40.3**	72.3 ± 13.4	77.8 ± 20.7
Diosmetin	78.3 ± 9.2	97.3 ± 33.0	**147.8 ± 13.8**
Clotrimazole	128.5 ± 12.3	76.7 ± 12.3	**207.8 ± 9.8**
Gliquidone	71.3 ± 14.7	79.1 ± 9.9	**241.8 ± 30.0**
Pararosaniline	**283.1 ± 10.2**	**194.9 ± 8.9**	102.8 ± 0.9
Compound 6	**196.6 ± 91.3**	**446.5 ± 98.2**	**121.9 ± 27.7**

OATP1B3-expressing CHO cells were preloaded for 2 h at room temperature with 10 nM [^3^H]-estradiol-17β-glucuronide. The potential OATP-substrates were added to yield final concentrations of 100, 10, or 1 µM and radioactivity remaining in the cells was measured after a 30-min incubation. Values are presented as % of control and represent the mean ± SD, of three independent experiments, each with quadruplicate determinations. Values in bold indicate significant differences to the control at *p* < 0.05. Compounds in italics are substrates; * compounds that are considered to be substrates.

For OATP1B3, the results are summarized in Table 2. In addition to the three neutral OATP1B1 compounds, berberine was neutral for OATP1B3. Similarly, as for OATP1B1, the mean of the neutral compounds was used as the control value for the statistics in Table 2. The most complete efflux was obtained with cyclosporine and salinomycin. These two compounds were the only ones that resulted in significant efflux at the 1 μM. Efflux initiated by bromocriptine, DIDS, and ketoconazole was similar at 10 μM and 100 μM. The next group of compounds, including clobetasol, β-estradiol 3-sulfate, progesterone, and indomethacin showed clear concentration dependency for all three concentrations. Eight compounds only resulted in efflux at the highest concentration of 100 μM. These were cilnidipine, naringin, verapamil, resveratrol, reserpine (*p* = 0.0654), clarithromycin (*p* = 0.0626), cimetidine, and gemfibrozil (*p* = 0.109). Finally, the last nine compounds produced unexpected efflux patterns. Like OATP1B1, gramicidin-mediated efflux was not concentration-dependent and only reached 30—40%. We saw the same pattern also with oleanolic acid. For nifedipine, efflux was more pronounced at 1 μM as compared to 10 μM. Ursolic acid triggered stimulation of uptake for estradiol-17β-glucuronide at 100 μM. Diosmetin, clotrimazole, and gliquidone stimulated uptake at 1 μM. Pararosaniline stimulated uptake at 10 μM and 100 μM. In contrast, compound 6 stimulated uptake at all three concentrations.

### Direct uptake of radiolabeled compounds

**FIGURE 3:**
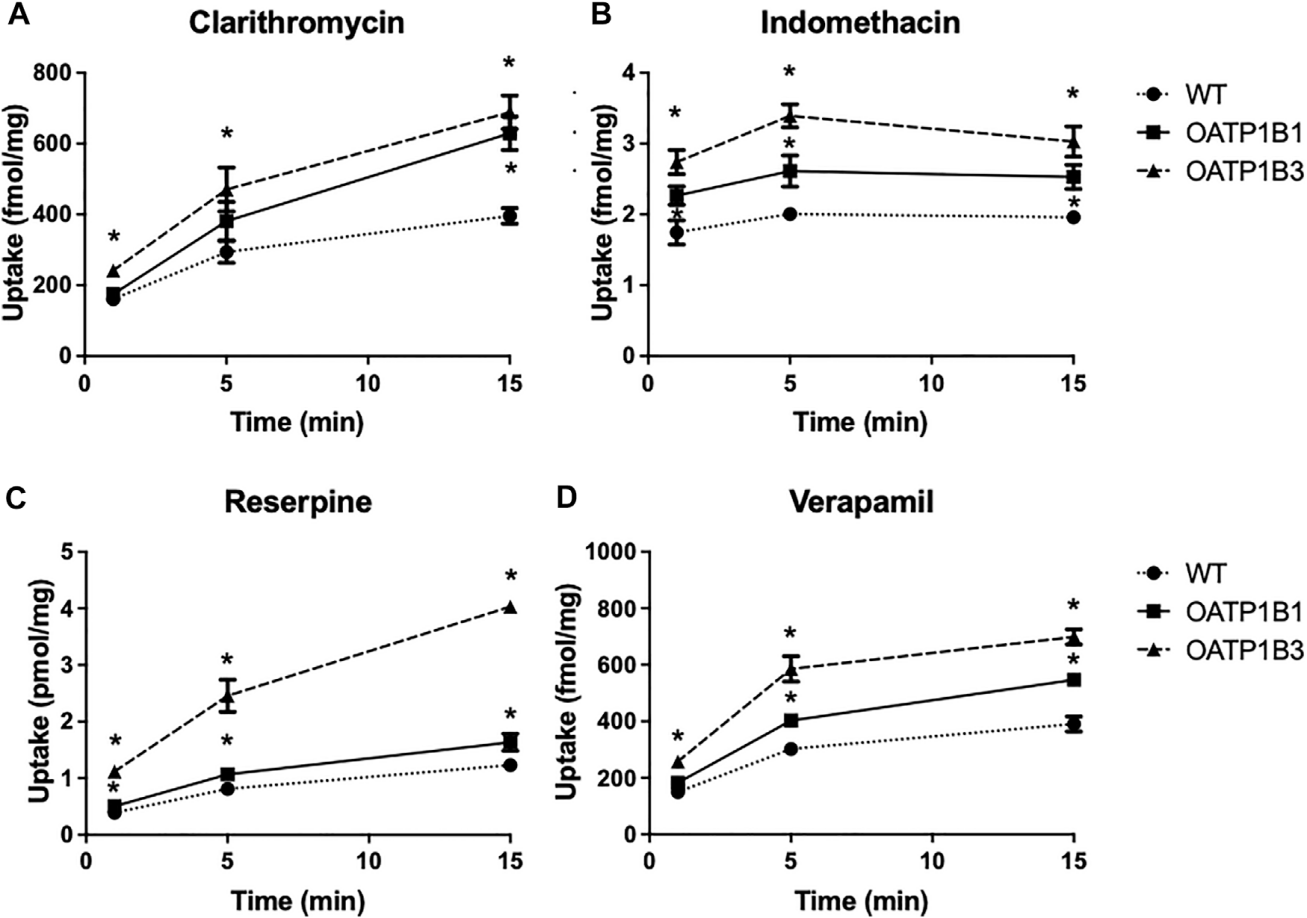
Time-dependent uptake of compounds predicted to be OATP substrates based on the CCF assay. Uptake of [^3^H]-clarithromycin (12.3 nM) **(A)**, [^3^H]-indomethacin (30 nM) **(B)**, [^3^H]-reserpine (15 nM) **(C)**, and [^3^H]-verapamil (1.8 nM) **(D)** was measured using CHO cells stably expressing OATP1B1 (squares), OATP1B3 (triangles), and wildtype CHO cells (circles) at 37°C on 24-well plates, and corrected for total protein. Two to three experiments were performed. The values (means ± SD) shown are from one representative experiment.

Based on the results in Tables 1 and 2 and the commercial availability of a tritiated version, we measured the uptake of anionic indomethacin and reserpine and cationic clarithromycin and verapamil using the OATP1B1- or OATP1B3-expressing CHO cells. The results are summarized in Figure 3 and demonstrate that all four compounds were transported in a time-dependent manner. Both OATPs transported all four compounds, but OATP1B3-mediated uptake was more substantial under the conditions used.

## Discussion

We successfully used the CCF assay for OATP1B1 and OATP1B3 by preloading the cells with the readily available common substrate [^3^H]-estradiol-17β-glucuronide. Except for pravastatin, the positive and negative controls behaved as expected (see below). We tested 30 compounds and identified 20 novel substrates for OATP1B1 (Table 1) and 16 for OATP1B3 (Table 2). This relatively high rate of identified substrates could indicate that most of the OATP1B inhibitors indeed are transported substrates and only few compounds are “only” inhibitors. Among these newly identified substrates are the cations berberine, clarithromycin, pararosaniline, and verapamil, the flavonoid naringin, antibiotic salinomycin, the non-steroidal anti-inflammatory drug (NSAID) indomethacin, the calcium channel blocker cilnidipine, the fibrate gemfibrozil, the steroids clobetasol and progesterone, the antifungals clotrimazole and ketoconazole, the blood pressure-lowering reserpine, and the anion exchange inhibitor DIDS. Based on the direct uptake of four novel compounds that are readily available in their radiolabeled form (clarithromycin, indomethacin, reserpine, and verapamil), we conclude that this assay works well. It can be used in the future to screen for and identify OATP1B1 or OATP1B3 substrates without the need to develop novel LC-MS/MS assays or the requirement of having the substrate in a radiolabeled form.

Based on the findings that OATP1B1 and OATP1B3 can transport pravastatin (Seithel et al., 2007; Kindla et al., 2011), we expected that pravastatin would lead to efflux of [^3^H]-estradiol-17β-glucuronide from both, OATP1B1- and OATP1B3-expressing cells. However, a concentration of 100 μM pravastatin only resulted in the efflux of [^3^H]-estradiol-17β-glucuronide in OATP1B1- but not in OATP1B3-expressing cells. Thus, the concentration of 100 μM might be too low to induce efflux. This agrees with the relatively higher K_m_ value published for OATP1B3 (228 μM) than OATP1B1 (104 μM). In addition, only SNPs in the Solute carrier of the OATP1B1 gene (*SLCO1B1*) have been associated with changes in pravastatin pharmacokinetics (Kivisto and Niemi, 2007), and pravastatin uptake in sandwich cultured human hepatocytes was associated with the expression of OATP1B1 but not with OATP1B3 (Kimoto et al., 2012).

Ouabain, one of the controls, was shown in *X. laevis* oocytes (Kullak-Ublick et al., 2001) and HEK293 cells (Gozalpour et al., 2014) to be a substrate of OATP1B3, but it was not transported by the respective OATP1B1-expressing system. Our efflux results confirm that it is only transported by OATP1B3 and not OATP1B1 (Figure 2).

Concerning the four compounds shown in Figure 3, all four undergo extensive first-pass metabolism in the liver, and thus uptake via OATP1B1 and OATP1B3 could contribute to this liver metabolism (Stitzel, 1976; Terhaag and Hermann, 1986; Fraschini et al., 1993; Rodvold, 1999).

It is interesting to note that we have identified novel organic cations as substrates for OATP1B1 and OATP1B3. Verapamil is known to have a high hepatic extraction resulting in a low oral bioavailability (Hamann et al., 1984; Echizen and Eichelbaum, 1986). One would assume that it is taken up into hepatocytes *via* OCT1. However, in a recent study where the CCF assay was established for human OCT1, verapamil did not exchange for any of the organic cations used to preload the cells. The conclusion was that verapamil is not a substrate of OCT1 (Boxberger et al., 2018). Thus, uptake via the OATPs could indeed be a significant uptake mechanism for its hepatic elimination. One study reported that OATPs contribute to the hepatic elimination of berberine, another cation that was identified as a substrate in our assay (Chen et al., 2015). Berberine uptake into rat hepatocytes was inhibited by rifampicin and cyclosporine A, known OATP inhibitors and substrates. Berberine was also a substrate of OATP1B3 when expressed in HEK293 cells. However, no uptake into OATP1B1-expressing HEK293 cells was observed (Chen et al., 2015). How can our contradicting findings that berberine can efflux [^3^H]-estradiol-17β-glucuronide from OATP1B1 but not from OATP1B3 cells be explained, given these direct uptake experiments? Inhibition of OATPs is substrate dependent, suggesting that the different substrates are transported *via* slightly different translocation pathways (Hagenbuch and Stieger, 2013). Thus, the fact that berberine does not exchange for [^3^H]-estradiol-17β-glucuronide in OATP1B3 points to a limitation of the CCF assay. A compound will only be identified as a substrate if it exchanges for the substrate used to preload the cells.

OATPs were initially characterized as transporters for organic anions. But already in 1996, it was demonstrated that OATP1A2 could transport the cation APD-ajmalinium (Bossuyt et al., 1996). Three years later, N-methylquinine, N-methylquinidine, and rocuronium, additional cations, were identified as substrates for OATP1A2 (van Montfoort et al., 1999). More recently, it was shown that the cation doxorubicin is a substrate of OATP1A2, OATP1B1, and OATP1B3 (Lee et al., 2017). Clarithromycin, verapamil, and berberine are additional organic cation substrates for OATP1B1 and OATP1B3, suggesting that other screens should be performed with these two OATPs, testing different organic cations that currently are considered to be taken up into hepatocytes *via* the organic cation transporters.

Cyclosporine is a substrate of ABCB1 (MDR1 or Pgp) (Saeki et al., 1993), and it has been extensively characterized as an OATP inhibitor. The CCF assay was also positive for cyclosporine, for which recently the trans-inhibition mechanism was further characterized (Izumi et al., 2022). The authors conclude, based on their experiments, that cyclosporine is not a substrate for OATP1B1. Thus, future experiments with radiolabeled cyclosporin that would allow using very low concentrations of substrate might be needed to clarify this issue. Our results indicate that cyclosporine has a very high affinity for OATP1B1 and OATP1B3, given that it was one of the most potent compounds resulting in efflux. This is in good agreement with the very low IC_50_ values that have been reported for the inhibition of both transporters in the sub-micromolar range and which were even lower after pre-incubation with cyclosporine (Gertz et al., 2013; Shitara and Sugiyama, 2017). Such increased inhibitor potency could be explained by a potential additional inhibition binding site on the cytoplasmic side of the membrane.

Bromocriptine is almost entirely metabolized in the liver (Maurer et al., 1983). In addition, it was suggested to be a substrate of OATP1B1 (Lu et al., 2006). Our findings confirm that it is an OATP1B1 substrate and show that it is also transported by OATP1B3, suggesting that uptake by the two liver-specific OATPs is at least in part the mechanism for its efficient liver metabolism.

Regarding compounds that resulted in unexpected efflux patterns, gramicidin, nifedipine, diosmetin, and gliquidone behaved similarly for both OATPs. Gramicidin is an unspecific ionophore that permits the rapid diffusion of potassium, protons, and alkali cations. The fact that it resulted in a consistent efflux of [^3^H]-estradiol-17β-glucuronide at each of the three concentrations and for both transporters indicates that it may interfere with the driving force of OATP-mediated transport. In the future, experiments with vesicles from OATP-expressing cells and gramicidin should be performed to elucidate this mechanism better. The increased uptake instead of efflux of [^3^H]-estradiol-17β-glucuronide in OATP1B3-expressing CHO cells due to nifedipine and gliquidone could indicate that they are substrates of OATP1B3 and inhibit an ABC efflux transporter whose activity is involved in the steady-state loading of [^3^H]-estradiol-17β-glucuronide. Nifedipine (Shukla et al., 2006), as well as gliquidone (Bell, 2017), are both substrates of BCRP (*ABCG2*) which is expressed in CHO-K1 cells (Gupta, 1988; Lebedeva et al., 2011). Thus, uptake via OATP1B3 could lead to inhibition of BCRP and thus to an increased accumulation of estradiol-17β-glucuronide. We speculate that the effects of clotrimazole, pararosaniline, and compound 6 are due to a similar mechanism, but additional experiments will be required to test this hypothesis. The negative control glucose resulted in a slight but significant increase in [^3^H]-estradiol-17β-glucuronide uptake rather than no effect. We assume that this has something to do with the still unknown transport mechanism of OATP1B-mediated uptake. It is clear that OATP1B1 works as an exchanger and the addition of glucose could have generated more of the normal physiological compound that exchanges for OATP1B1-mediated substrate uptake. Further experiments are required to elucidate this finding in more detail.

In summary, we have used the CCF assay for OATP1B1 and OATP1B3 with [^3^H]-estradiol-17β-glucuronide as the tracer substrate. We identified several novel substrates for both OATPs, particularly the additional organic cations, clarithromycin, and verapamil. Future screens, e.g., for anticancer drugs, could be performed with this assay to identify which of the many drugs that interact with OATP1B1 and OATP1B3 are transported and pose a risk for potential liver toxicity.

## Data Availability

The original contributions presented in the study are included in the article/Supplementary Material, further inquiries can be directed to the corresponding author.

## References

[B35] Bell S. D. (2017). Understanding pregnancy centered medications: Characterizing the interactions of a series of Sulfonylurea Analogs and the ATP binding Cassette transporter proteins, P-Glycoprotein and breast cancer resistance protein. Open Access Dissertations Paper 747. 10.23860/diss-bell-samuel-2017

[B1] Bossuyt X. Muller M. Meier P. J. (1996). Multispecific amphipathic substrate transport by an organic anion transporter of human liver. J. Hepatol. 25 (5), 733–738. 10.1016/s0168-8278(96)80246-7 8938553

[B2] Boxberger K. H. Hagenbuch B. Lampe J. N. (2018). Ligand-dependent modulation of hOCT1 transport reveals discrete ligand binding sites within the substrate translocation channel. Biochem. Pharmacol. 156, 371–384. 10.1016/j.bcp.2018.08.028 30138624 PMC6195816

[B3] Chen C. Wu Z. T. Ma L. L. Ni X. Lin Y. F. Wang L. (2015). Organic anion-transporting polypeptides contribute to the hepatic uptake of berberine. Xenobiotica. 45 (12), 1138–1146. 10.3109/00498254.2015.1042537 26068524

[B4] De Bruyn T. van Westen G. J. Ijzerman A. P. Stieger B. de Witte P. Augustijns P. F. (2013). Structure-based identification of OATP1B1/3 inhibitors. Mol. Pharmacol. 83 (6), 1257–1267. 10.1124/mol.112.084152 23571415

[B5] Echizen H. Eichelbaum M. (1986). Clinical pharmacokinetics of verapamil, nifedipine and diltiazem. Clin. Pharmacokinet. 11 (6), 425–449. 10.2165/00003088-198611060-00002 3542336

[B6] Fraschini F. Scaglione F. Demartini G. (1993). Clarithromycin clinical pharmacokinetics. Clin. Pharmacokinet. 25 (3), 189–204. 10.2165/00003088-199325030-00003 8222460

[B7] Gertz M. Cartwright C. M. Hobbs M. J. Kenworthy K. E. Rowland M. Houston J. B. (2013). Cyclosporine inhibition of hepatic and intestinal CYP3A4, uptake and efflux transporters: Application of PBPK modeling in the assessment of drug-drug interaction potential. Pharm. Res. 30 (3), 761–780. 10.1007/s11095-012-0918-y 23179780

[B8] Gozalpour E. Greupink R. Wortelboer H. M. Bilos A. Schreurs M. Russel F. G. (2014). Interaction of digitalis-like compounds with liver uptake transporters NTCP, OATP1B1, and OATP1B3. Mol. Pharm. 11 (6), 1844–1855. 10.1021/mp400699p 24754247

[B9] Gui C. Miao Y. Thompson L. Wahlgren B. Mock M. Stieger B. (2008). Effect of pregnane X receptor ligands on transport mediated by human OATP1B1 and OATP1B3. Eur. J. Pharmacol. 584 (1), 57–65. 10.1016/j.ejphar.2008.01.042 18321482 PMC2376123

[B10] Gupta R. S. (1988). Intrinsic multidrug resistance phenotype of Chinese hamster (rodent) cells in comparison to human cells. Biochem. Biophys. Res. Commun. 153 (2), 598–605. 10.1016/s0006-291x(88)81137-9 3382391

[B11] Hagenbuch B. Stieger B. (2013). The SLCO (former SLC21) superfamily of transporters. Mol. Asp. Med. 34 (2-3), 396–412. 10.1016/j.mam.2012.10.009 PMC3602805 23506880

[B12] Hamann S. R. Blouin R. A. McAllister R. G. Jr. (1984). Clinical pharmacokinetics of verapamil. Clin. Pharmacokinet. 9 (1), 26–41. 10.2165/00003088-198409010-00002 6362951

[B13] Harper J. N. Wright S. H. (2013). Multiple mechanisms of ligand interaction with the human organic cation transporter, OCT2. Am. J. Physiol. Ren. Physiol. 304 (1), F56–F67. 10.1152/ajprenal.00486.2012 PMC3971011 23034939

[B14] Izumi S. Nozaki Y. Lee W. Sugiyama Y. (2022). Experimental and modeling evidence supporting the trans-inhibition mechanism for preincubation time-dependent, long-lasting inhibition of organic anion transporting polypeptide 1B1 by cyclosporine A. Drug Metab. Dispos. 50 (5), 541–551. 10.1124/dmd.121.000783 35241487

[B15] Karlgren M. Vildhede A. Norinder U. Wisniewski J. R. Kimoto E. Lai Y. (2012). Classification of inhibitors of hepatic organic anion transporting polypeptides (OATPs): Influence of protein expression on drug-drug interactions. J. Med. Chem. 55 (10), 4740–4763. 10.1021/jm300212s 22541068 PMC3361267

[B16] Kimoto E. Yoshida K. Balogh L. M. Bi Y. A. Maeda K. El-Kattan A. (2012). Characterization of organic anion transporting polypeptide (OATP) expression and its functional contribution to the uptake of substrates in human hepatocytes. Mol. Pharm. 9 (12), 3535–3542. 10.1021/mp300379q 23082789

[B17] Kindla J. Muller F. Mieth M. Fromm M. F. Konig J. (2011). Influence of non-steroidal anti-inflammatory drugs on organic anion transporting polypeptide (OATP) 1B1- and OATP1B3-mediated drug transport. Drug Metab. Dispos. 39 (6), 1047–1053. 10.1124/dmd.110.037622 21389119

[B18] Kivisto K. T. Niemi M. (2007). Influence of drug transporter polymorphisms on pravastatin pharmacokinetics in humans. Pharm. Res. 24 (2), 239–247. 10.1007/s11095-006-9159-2 17177112

[B19] Kullak-Ublick G. A. Ismair M. G. Stieger B. Landmann L. Huber R. Pizzagalli F. (2001). Organic anion-transporting polypeptide B (OATP-B) and its functional comparison with three other OATPs of human liver. Gastroenterology 120 (2), 525–533. 10.1053/gast.2001.21176 11159893

[B20] Lebedeva I. V. Pande P. Patton W. F. (2011). Sensitive and specific fluorescent probes for functional analysis of the three major types of mammalian ABC transporters. PLoS One 6 (7), e22429. 10.1371/journal.pone.0022429 21799851 PMC3142157

[B21] Lee H. H. Leake B. F. Kim R. B. Ho R. H. (2017). Contribution of organic anion-transporting polypeptides 1A/1B to doxorubicin uptake and clearance. Mol. Pharmacol. 91 (1), 14–24. 10.1124/mol.116.105544 27777271 PMC5198512

[B22] Lu W. J. Huang K. Lai M. L. Huang J. D. (2006). Erythromycin alters the pharmacokinetics of bromocriptine by inhibition of organic anion transporting polypeptide C-mediated uptake. Clin. Pharmacol. Ther. 80 (4), 421–422. 10.1016/j.clpt.2006.06.003 17015059

[B23] Maurer G. Schreier E. Delaborde S. Nufer R. Shukla A. P. (1983). Fate and disposition of bromocriptine in animals and man. II: Absorption, elimination and metabolism. Eur. J. Drug Metab. Pharmacokinet. 8 (1), 51–62. 10.1007/BF03189581 6861794

[B24] Rodvold K. A. (1999). Clinical pharmacokinetics of clarithromycin. Clin. Pharmacokinet. 37 (5), 385–398. 10.2165/00003088-199937050-00003 10589373

[B25] Saeki T. Ueda K. Tanigawara Y. Hori R. Komano T. (1993). Human P-glycoprotein transports cyclosporin A and FK506. J. Biol. Chem. 268 (9), 6077–6080. 10.1016/s0021-9258(18)53221-x 7681059

[B26] Schafer A. M. Bock T. Meyer Zu Schwabedissen H. E. (2018). Establishment and validation of competitive counterflow as a method to detect substrates of the organic anion transporting polypeptide 2B1. Mol. Pharm. 15 (12), 5501–5513. 10.1021/acs.molpharmaceut.8b00631 30380886

[B27] Schafer A. M. Gilgen P. M. Spirgi C. Potterat O. Meyer Zu Schwabedissen H. E. (2022). Constituents of passiflora incarnata, but not of valeriana officinalis, interact with the organic anion transporting polypeptides (OATP)2B1 and OATP1A2. Planta Med. 88 (2), 152–162. 10.1055/a-1305-3936 33511622

[B28] Seithel A. Eberl S. Singer K. Auge D. Heinkele G. Wolf N. B. (2007). The influence of macrolide antibiotics on the uptake of organic anions and drugs mediated by OATP1B1 and OATP1B3. Drug Metab. Dispos. 35 (5), 779–786. 10.1124/dmd.106.014407 17296622

[B29] Shitara Y. Sugiyama Y. (2017). Preincubation-dependent and long-lasting inhibition of organic anion transporting polypeptide (OATP) and its impact on drug-drug interactions. Pharmacol. Ther. 177, 67–80. 10.1016/j.pharmthera.2017.02.042 28249706

[B30] Shukla S. Robey R. W. Bates S. E. Ambudkar S. V. (2006). The calcium channel blockers, 1, 4-dihydropyridines, are substrates of the multidrug resistance-linked ABC drug transporter, ABCG2. Biochemistry 45 (29), 8940–8951. 10.1021/bi060552f 16846237

[B31] Stitzel R. E. (1976). The biological fate of reserpine. Pharmacol. Rev. 28 (3), 179–208. 16280

[B32] Terhaag B. Hermann U. (1986). Biliary elimination of indomethacin in man. Eur. J. Clin. Pharmacol. 29 (6), 691–695. 10.1007/BF00615960 3709611

[B33] van Montfoort J. E. Hagenbuch B. Fattinger K. E. Muller M. Groothuis G. M. Meijer D. K. (1999). Polyspecific organic anion transporting polypeptides mediate hepatic uptake of amphipathic type II organic cations. J. Pharmacol. Exp. Ther. 291 (1), 147–152. 10490898

[B34] Zhang Y. Hays A. Noblett A. Thapa M. Hua D. H. Hagenbuch B. (2013). Transport by OATP1B1 and OATP1B3 enhances the cytotoxicity of epigallocatechin 3-O-gallate and several quercetin derivatives. J. Nat. Prod. 76 (3), 368–373. 10.1021/np3007292 23327877 PMC3606651

